# High-Performance Bipolar Membrane Development for Improved Water Dissociation

**DOI:** 10.1021/acsapm.0c00653

**Published:** 2020-08-19

**Authors:** Yingying Chen, Jacob A. Wrubel, W. Ellis Klein, Sadia Kabir, Wilson A. Smith, K. C. Neyerlin, Todd G. Deutsch

**Affiliations:** † National Renewable Energy Laboratory, Golden, Colorado 80401, United States; ‡ Department of Chemical and Biological Engineering, University of Colorado Boulder, Boulder, Colorado 80303, United States; § Renewable and Sustainable Energy Institute (RASEI), University of Colorado Boulder, Boulder, Colorado 80303, United States

**Keywords:** bipolar membranes, graphene oxide, electrospun nanofibers, three-dimensional junction, flow cell, AC impedance

## Abstract

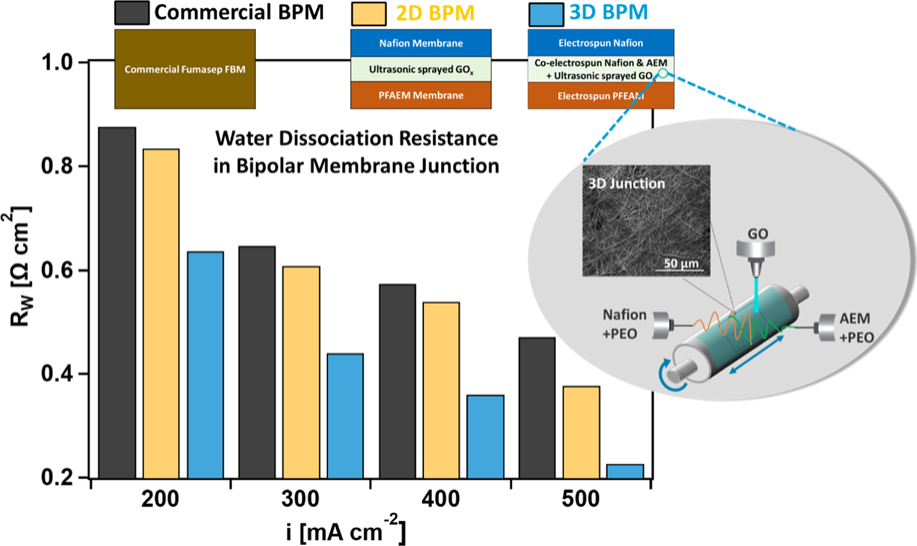

Bipolar membranes (BPMs) are the enabling component of many promising electrochemical devices used for separation and energy conversion. Here, we describe the development of high-performance BPMs, including two-dimensional BPMs (2D BPMs) prepared by hot-pressing two preformed membranes and three-dimensional BPMs (3D BPMs) prepared by electrospinning ionomer solutions and polyethylene oxide. Graphene oxide (GO_*x*_) was introduced into the BPM junction as a water-dissociation catalyst. We assessed electrochemical performance of the prepared BPMs by voltage–current (*V*–*I*) curves and galvanostatic electrochemical impedance spectroscopy. We found the optimal GO_*x*_ loading in 2D BPMs to be 100 μg cm^–2^, which led to complete coverage of GO_*x*_ at the interface. The integration of GO_*x*_ beyond this loading moderately improved electrochemical performance but significantly compromised mechanical strength. GO_*x*_-catalyzed 2D BPMs showed comparable performance with a commercially available Fumasep BPM at current densities up to 500 mA cm^–2^. The 3D BPMs exhibited even better performance: lower resistance and higher efficiency for water dissociation and substantially higher stability under repeated cycling up to high current densities. The improved electrochemical performance and mechanical stability of the 3D BPMs make them suitable for incorporation into CO_2_ electrolysis devices where high current densities are necessary.

## 1 Introduction

Bipolar membranes (BPMs) have been studied for a wide variety of applications for decades. Traditionally, they are mostly used in electrodialysis stacks for producing acids and bases at each side of the BPMs for different applications, such as the recovery of organic acids from fermentation broths,^1,2^ pH control in biochemical processes,^3^ water desalination,^4,5^ and deacidification of fruit juices.^6^ More recently, membrane electrode assemblies (MEAs) that incorporate BPMs have attracted considerable interest for applications such as CO_2_ reduction (CO_2_R) electrolyzers,^7−10^ fuel cells,^11−13^ and water electrolyzers.^14^ In water electrolyzers with BPM MEAs, a pH gradient is established for providing a favorable environment for electrochemical reactions at their respective electrodes. The oxygen evolution reaction (OER) occurs at the alkaline anode, while the hydrogen evolution reaction occurs at the acidic cathode, environments with facile kinetics that permit low-platinum loading or nonplatinum electrocatalysts.^15−17^ In CO_2_R electrolyzers, a BPM not only enables alkaline OER for nonprecious electrocatalysts but also significantly decreases the crossover of carbonate and CO_2_R products to improve the device efficiency and stability compared to CO_2_R electrolyzers with monopolar membranes.^18^

A BPM typically consists of three layers: a strong acid cation-exchange layer (CEL), a strong base anion-exchange layer (AEL), and a junction layer in between, which usually contains a catalyst that promotes water dissociation. Under reverse-bias polarization, the CEL faces the cathode and the AEL faces the anode. Salt ions migrate away from the junction, and charge is carried by the dissociation of water molecules into H^+^ and OH^–^. The H^+^ and OH^–^ ions migrate toward the respective electrodes through the ion-exchange layers.

Water dissociation in a BPM junction can be up to 50 million times faster than in an aqueous solution,^19^ which involves two factors. One factor is that catalysts in the BPM junction provide a surface that presents an alternative path for water dissociation, and therefore, it decreases the activation energy; this is referred to as the chemical reaction mechanism (CRM). Instead of direct water dissociation, shown as
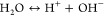
1reversible protonation–deprotonation reactions occur between water molecules and weakly acidic/basic catalyst sites:^20−23^

With a weak-acid catalyst A
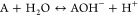
2
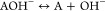
3

With weak-base catalyst B
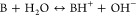
4
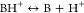
5

Common catalysts used in most commercial BPMs and those reported in literature are either weak acids or weak bases with a p*K*_a_ between 4 and 10, such as tertiary amines,^24^ carboxylic acids,^25^ phosphoric acid,^26^ and metal hydroxides.^27−29^ Graphene oxide (GO_*x*_) has been recognized as a more efficient catalyst for water dissociation in BPMs^30−32^ because of its large specific area and abundant oxygen-containing hydroxyl, carboxyl, carbonyl, and epoxide^33,34^ functional groups.

The other phenomenon contributing to the promoted water dissociation in a BPM junction is the electric-field enhancement effect or second Wien effect (SWE).^35^ The electric field across the thin junction could be as high as 10^8^ to 10^9^ V m^–1^ under reverse bias, which can increase the water dissociation rate by 6–7 orders of magnitude compared to that in the absence of an electric field.^35−37^

When incorporated in MEA devices, BPMs are expected to experience high current densities (>500 mA cm^–2^) and even forward bias, which could potentially cause accelerated degradation. The major requirements for a well-designed BPM for these applications may include (a) high water-dissociation efficiency, (b) sufficient water transport into the BPM junction to prevent dehydration at high current densities, and (c) high mechanical stability to prevent delamination/blistering at high current densities or forward bias. However, most BPMs are fabricated by pressing preformed layers or casting, which leads to an interface that has insufficient bonding between the layers to prevent membrane delamination. Shen and co-workers^28^ observed that the commercial BPM Fumasep-FBM underwent irreversible damage at reverse bias current densities above 600 mA cm^–2^.

In the past decade, electrospinning for membrane fabrication has drawn great attention. Electrospinning yields polymer fibers that are at a submicrometer scale, where an increase in contact area can lead to more robust mechanical properties, enhanced ionic conductivities, and improved performance. Several studies^38−40^ have used electrospinning to make high-performance Nafion polymers and functionalized Nafion polymers for fuel cells, whereas fewer studies have focused on fabricating anion-exchange membranes. Park and co-authors^41^ managed to fabricate a robust AEM with very high ionic conductivity and ion-exchange capacity while still maintaining a controlled swelling ratio and good mechanical strength. However, even though many studies have reported a variety of methods for BPM fabrication, there have been only a few studies reported on electrospun BPMs. In 2017, Pan et al.^42^ reported BPM preparation via electrospinning followed by hot-pressing. They tested BPMs catalyzed with polyethylene glycol (PEG) in the junction and observed a much lower potential drop compared with those that had no PEG whether the BPMs had been electrospun or made by casting. More remarkable was the voltage necessary to achieve 100 mA cm^–2^ in the best electrospun junction was only ∼2.25 V compared with ∼9 V for the best BPM made by casting. Shen et al.^28^ proposed a novel electrospun BPM with a three-dimensional (3D) junction made by dual-fiber electrospinning to increase the interpenetration and contact points between the different polymers, which showed no evident damage at current densities up 1.2 A cm^–2^. Electrospun BPMs provide structural advantages for both water dissociation performance and membrane durability, as has been reported by Pan et al.^42^ and Shen et al.^28^ However, few studies have quantitatively compared the water dissociation resistance between electrospun BPMs and BPMs made by other approaches. In addition, with GO_*x*_ emerging as one of the most promising catalysts for water dissociation in BPMs in recent years,^30−32^ no previous study has incorporated GO_*x*_ into electrospun BPMs.

Here, we present an electrospun 3D BPM with a dual-fiber co-electrospun junction with GO_*x*_ sprayed (concurrently during electrospinning) between the fibers to act as a catalyst for water dissociation. The intertwined fibers with GO_*x*_ applied on their surface provide a substantially higher catalytic area for the reaction compared to a planar junction. Two-dimensional BPMs (2D BPMs) with a planar junction were also fabricated, and the effect of GO_*x*_ loading in the junction was extensively investigated using voltage–current (*V*–*I*) measurements and electrochemical impedance spectroscopy (EIS). The performance of 2D BPMs and 3D BPMs with the same catalyst loading was compared to a commercial BPM at current densities up to 500 mA cm^–2^. This is the first study to undertake a quantitative analysis of water dissociation via EIS in electrospun BPMs and compare it to 2D BPMs and commercial BPMs. The stability of the BPMs was assessed by the voltage changes during repeated galvanodynamic scans and long-term galvanostatic holding at 500 mA cm^–2^. Our results demonstrate that 3D BPMs showed better stability and lower water-dissociation resistance than 2D BPMs and commercial BPMs.

## 2 Methods

### 2.1 Materials

Nafion membranes (NR211) with a thickness of 25 μm and Nafion ionomer dispersion D2020 (1000 EW, 20 wt %) were purchased from Ion Power. Perfluorinated anion-exchange ionomer and membranes (PFAEMs) were synthesized in-house; the synthesis and fabrication procedures have been described previously.^43^ GO_*x*_ paste with a concentration of 30–35 g L^–1^ was commercially obtained from Graphene Supermarket and diluted to 10 g L^–1^ with 18 MΩ cm deionized (DI) water. According to the manufacturer, the GO_*x*_ composition is 79% C and 20% O and the flake size is around 0.5–5 μm. Poly(ethylene oxide) (PEO) of 400 kDa MW was obtained from Sigma-Aldrich. The commercial BPM Fumasep FBM (Fumatech GmbH, Germany), obtained from Fuel Cell Store, is composed of a sulfonated cross-linked poly-ether ether ketone and is used as the CEL; polysulfone with bicyclic amines is used as the AEL, with a polyacrylic acid/polyvinyl pyridine salt complex in the junction.^44^ Isopropanol alcohol (IPA) of HPLC grade (99.8%) was purchased from Sigma-Aldrich. NaOH (1 M) was made by dissolving NaOH pellets (Certified ACS, from Fisher Chemical) in DI water, and 1 M H_2_SO_4_ was made by diluting 95.0–98.0 w/w % H_2_SO_4_ (Certified ACS Plus, from Fisher Chemical) with DI water.

### 2.2 Water Uptake Measurement

Water uptake was measured with dynamic vapor sorption (DVS, TA Instruments Q5000). A dry film sample weighing 3–6 mg was first loaded into the DVS and preconditioned at 0% relative humidity (RH) and 60 °C for 3 h. Only a small weight loss (<5%) was observed during this 3 h period, and the mass equilibrated for at least 1 h before the end of the 3 h drying step. The RH was then systematically changed to constant values for 3 h each at a fixed temperature of 60 °C. Again, the mass equilibrated at least 1 h before the end of each 3 h step. The water uptake in the membrane was calculated according to eq 6
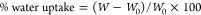
6where *W*_0_ and *W* are dry and wet membrane weights measured at the end of the drying step and at the end of each humidified step, respectively.

### 2.3 Fabrication of 2D BPMs

**Figure 1:**
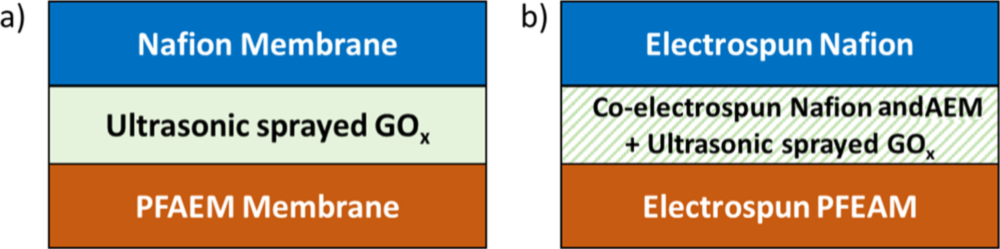
Structure of (a) 2D BPMs and (b) 3D BPMs.

Two-dimensional BPMs with different junctions were prepared by hot-pressing together Nafion and PFAEM films together. A mixture of GO_*x*_ and Nafion was ultrasonically sprayed on a Nafion membrane laid atop a heated vacuum table at 80 °C using an ultrasonic AccuMist spray nozzle (Sono-Tek Exacta-Coat). The flow rate was controlled by a syringe pump to be at a constant rate of 0.3 mL min^–1^. The sprayed ink was composed of a 1:1 ratio of GO_*x*_ and Nafion, and a 1:1 ratio of DI water and IPA. Nafion membranes with GO_*x*_ deposition were hot-pressed at 120 °C and 3.38 MPa for 2 min to anneal the catalyst layer. Two-dimensional BPMs were formed by hot-pressing the Nafion with GO_*x*_ deposition and a PFAEM (∼30 μm) together at 60 °C, 3.38 MPa for 2 min. Before hot-pressing, the Nafion and AEM layers were wetted in DI water and then spread together. A Gylon sheet was used to gently squeeze out bubbles and void space between them. The BPMs were stored in DI water prior to testing. The structure of the 2D BPMs is shown in Figure 1a.

A similar method of preparation was followed for the control 2D BPM. In this case, the deposition of GO_*x*_ and Nafion spray was left out, resulting in an uncatalyzed 2D BPM.

### 2.4 Fabrication of 3D BPMs

The electrospinning ink was made by mixing an ionomer dispersion and PEO in a DI water and IPA mixture. PEO serves as the carrier polymer.^38^ In the Nafion ink, polymer solids accounted for 15 wt %, in which a Nafion and PEO ratio of 99:1 was used. For the AEM ink, an AEM and PEO ratio of 98:2 was used in 10 wt % solid. The ink was homogenized on a drum mixer overnight before electrospinning.

Electrospinning was conducted using a custom-built electrospinning apparatus that has dual-fiber electrospinning capabilities. The grounded collection drum can move simultaneously in two axes—rotation around the center axis and linear oscillation along the center axis—to ensure a random distribution and orientation of fibers on the drum. The electrospinning conditions for Nafion fibers and AEM fibers are listed in Table 1. All electrospinning processes were performed at room temperature.

**Table 1:** Optimized Process Parameters for Electrospinning Nafion and PFAEM Fibers

ionomer	tip-to-collector distance (cm)	RH (%)	flow rate (mL h^–1^)	applied voltage (kV)
Nafion	8	30	0.2	10
PFAEM	6	30	0.3	5

To make a 3D BPM, a layer of Nafion fiber was electrospun on the drum. On top of that, a 3D junction was made by co-electrospinning Nafion fibers and AEM fibers from two needles, one on each side of the drum, while 2 mg mL^–1^ GO_*x*_ with 1:1 ratio of Nafion was sprayed from an ultrasonic AccuMist spray nozzle (Sono-Tek Exacta-Coat) on top of the drum at 0.1 mL min^–1^. Electrospinning from both sides and catalyst spraying were performed simultaneously for 1 min followed by a 1 min dwell. The cumulative spraying time was 60 min, leading to a final loading of 100 μg cm^–2^ GO_*x*_. A similar method of co-electrospun 3D junction fabrication was reported by Shen et al.;^28^ however, they used 1.5 mg cm^–2^ Al(OH)_3_ as the water dissociation catalyst, where we have used GO_*x*_. At the end, a layer of the AEM fiber mat was electrospun on top of the 3D junction. The electrospun fiber mat was densified by exposing it to IPA vapor at room temperature for 15 min on each side, followed by hot-pressing at 60 °C and 3.38 MPa for 2 min. A similar BPM fiber mat post-treatment was used by Pan et al.^42^ The structure of the 3D BPMs is shown in Figure 1b.

### 2.5 Flow Cell Testing

**Figure 2:**
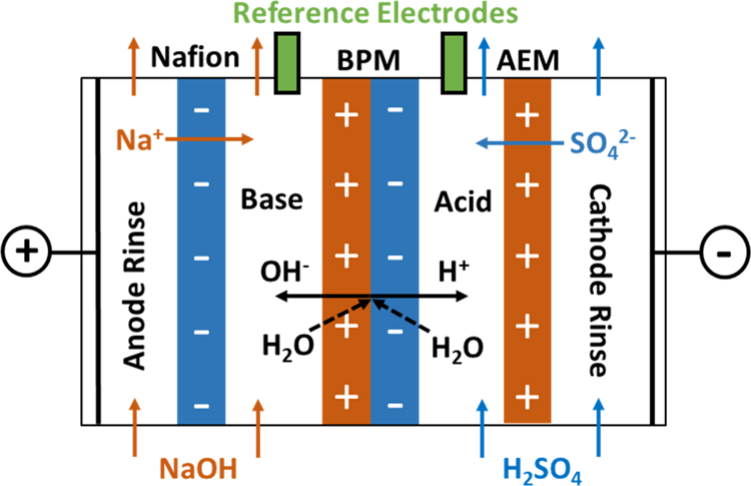
Schematic diagram of the flow cell.

The electrochemical characterization of the membranes was investigated in a tailor-made four-chamber (anode rinse, base, acid, and cathode rinse) flow cell shown in Figure 2. A 1 M NaOH solution was fed to the anode rinse chamber and base chamber at 10 mL min^–1^, and 1 M H_2_SO_4_ was fed to the cathode rinse chamber and acid chamber at 10 mL min^–1^. Two pieces of Pt foil (99.99%, 0.001 in.-thick, Alfa Aesar) supported on Ti plates were used as the working and counter electrodes. The BPM was placed at the center of the cell with an AEM (Neosepta AHA) and a CEM (Nafion NR-212) at each side to minimize the influence of the electrode reaction on measurements. The effective BPM area was defined using a 1.2 cm-diameter circular aperture. All the measurements were conducted at room temperature.

The potential difference across the membrane was measured by a Hg/Hg_2_SO_4_ reference electrode [filled with saturated K_2_SO_4_, *E*^0^ = 0.64 V vs normal hydrogen electrode (NHE) at 25 °C] in the acidic chamber and a Hg/HgO reference electrode (filled with 1 M NaOH, *E*^0^ = 0.098 V vs NHE at 25 °C) in the alkaline chamber. Chronopotentiometry and galvanostatic EIS of BPMs were conducted at room temperature using a Gamry Reference 3000 potentiostat in a standard four-electrode setup. In EIS measurements, an AC amplitude of 10% of the applied DC current and a frequency spectrum from 300 kHz to 1 Hz was employed.

### 2.6 Morphology

Morphological characterization of the nanofibers and BPMs was done using an environmental scanning electron microscopy (ESEM, FEI Quanta 60).

## 3 Results and Discussion

### 3.1 BPM Development and Characterization

The BPMs studied here include a 2D BPM prepared by hot-pressing together two preformed membranes and a 3D BPM prepared by electrospinning with GO_*x*_ as the catalyst in a dual-fiber co-electrospun junction. The cation-exchange ionomer Nafion and anion-exchange ionomer PFAEM were used in fabricating both 2D BPMs and 3D BPMs, with their relevant ionic and electronic properties listed in Table 2. Nafion ionomers have been a mature product in the market for more than 30 years, widely used in fuel cells and water electrolyzers in industrial and academic research.^45^ Nafion shows high permselectivity for cations and high stability both mechanically and chemically. As proposed by McDonald,^46^ Nafion is an attractive CEL in BPM fabrication for photoelectrochemical conversion applications. The PFAEM used in this study was first reported by Park et al. and has demonstrated high conductivity and high chemical durability against hydroxide attack.^43^ Using these two perfluorinated ionomers in the BPM fabrication processes reported in this study provides intrinsically high conductivity and durability to the membranes. In addition, both PFAEM and Nafion ionomers have similar solubility in IPA. When the 3D BPM fiber mat is exposed to the IPA vapor during post-treatment, the ionomer fibers of opposite charges would both be slightly dissolved, forming a large contact area.

**Table 2:** Characteristics of Cation-Exchange and Anion-Exchange Ionomer Used in the Fabrication of 2D BPM and 3D BPM

polymer	ionic form	IEC (mmol/g)	conductivity (mS/cm)	expansion ratio (%)	water uptake (%)
Nafion	H^+^	0.92	∼70^47^	10	25^a^
PFAEM	Cl^–^	1.02	43^43^	16	38^b^

a Measured from dry membrane to conditioned in water at 60 °C.^48^

b Measured from dry membrane to conditioned in 95% humidity at 60 °C.^43^

**Figure 3:**
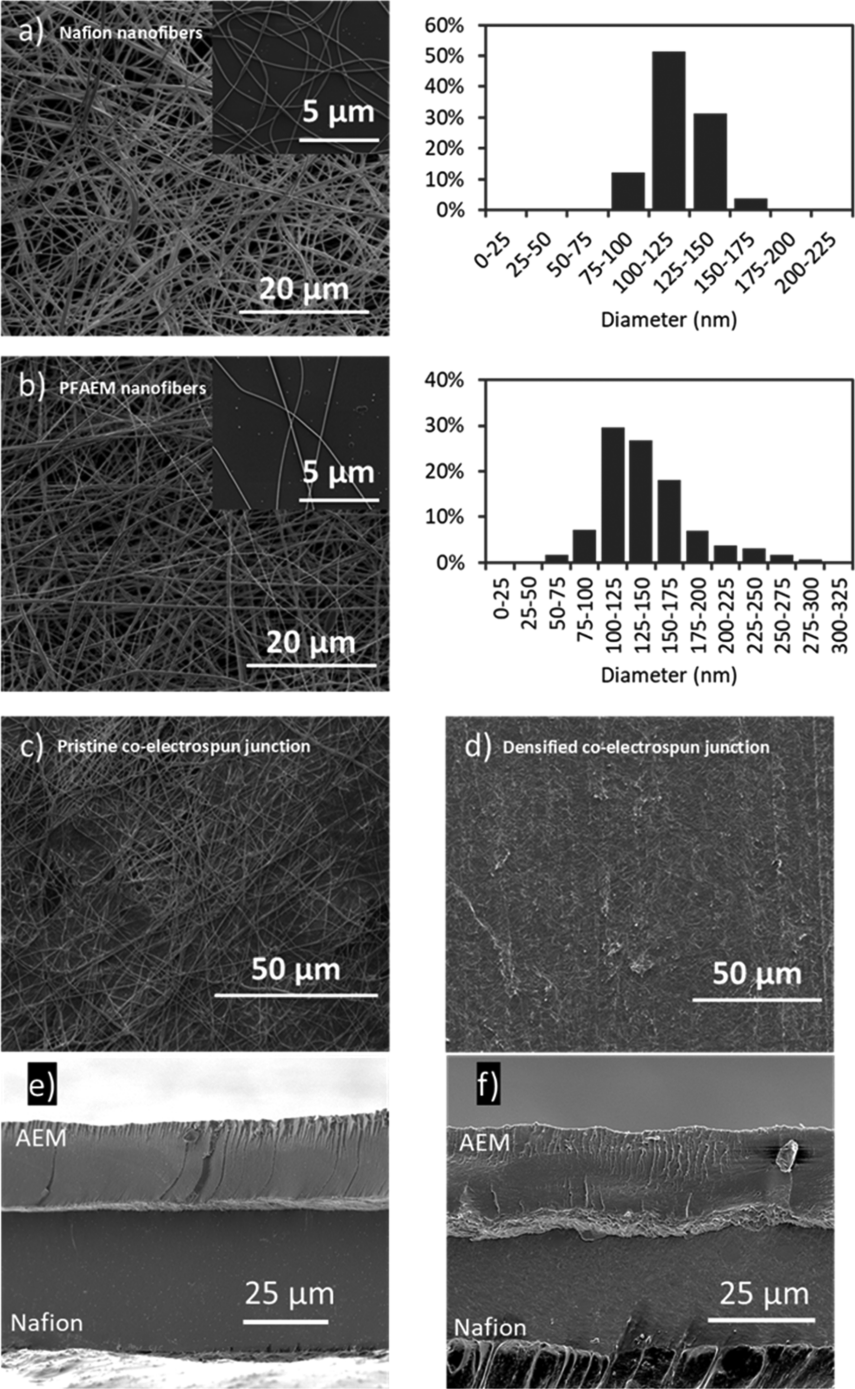
SEM images of (a) Nafion fibers and (b) PFAEM fibers and the corresponding fiber diameters measured with ImageJ software; (c) pristine and (d) densified 3D junction; (e) cross section of 2D BPM with 100 μg cm^–2^ GO in junction; and (f) cross section of 3D BPM with 100 μg cm^–2^ GO in the co-electrospun 3D junction.

The electrospun BPMs are composed of three layers: a Nafion fiber layer, a catalyzed 3D-junction co-electrospun layer, and a PFAEM fiber layer. The morphology of the Nafion and PFAEM fibers electrospun using the conditions listed in Table 1 were observed using an scanning electron microscope (Figure 3). The fiber diameters were measured with ImageJ software. Uniform and defect-free fibers were observed for both Nafion and PFAEM, with an average diameter of 112.5 ± 12.5 nm for the Nafion fibers, and 125 ± 25 nm for the PFAEM fibers (averaged over 5000+ measurements for the Nafion fibers and 20,000+ measurements for the PFAEM fibers). In fabricating the 3D junction, the mixture of GO_*x*_ and Nafion with DI and IPA as solvents was sprayed during the co-electrospinning of Nafion fibers and PFAEM fibers. The co-electrospun junction exhibited a denser structure (Figure 3c) than the individual fiber mats. The GO_*x*_ and Nafion mixture infills voids between the fibers, leading to nearly full catalyst coverage and better contact between fibers of opposite fixed charge. After exposure of the 3D junction to the solvent vapor and hot-pressing, a densified structure with fibers fused together was observed (Figure 3d). The thickness of the Nafion and PFAEM layers in the densified 3D BPM was about 30 μm each. The thickness of the 3D junction was ∼3 to 5 μm, accounting for ∼5 to 8% of the thickness of the individual membrane. The total thickness of ∼60 μm effectively reduced co-ion leakage through the membranes. With a similar thickness of 2D BPM and 3D BPM, it allows for a fair comparison of the performance between two kinds of membranes fabricated with different approaches. SEM images of the cross section of the 2D BPM and 3D BPM are shown in Figure 3e,f, respectively.

**Figure 4:**
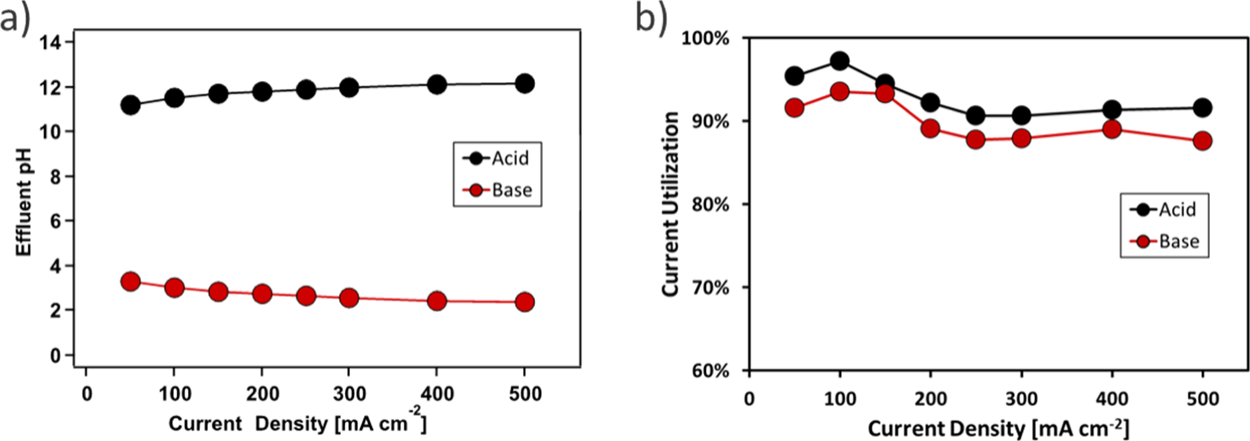
(a) Effluent pH and (b) respective current utilization from the acid chamber (next to CEL of BPM) and base chamber (next to AEL of BPM) as a function of the current density. 0.4 M K_2_SO_4_ was fed to the acid and base chamber at 10 mL min^–1^. KOH (1 M) was fed to the anode rinse and 1 M H_2_SO_4_ was fed to the cathode rinse at 10 mL min^–1^. Three-dimensional BPM with 100 μg cm^–2^ GO_*x*_ was used.

The effluent pH shown in Figure 4a was measured from both sides of the electrospun BPM when flowing 0.4 M K_2_SO_4_ solution of neutral pH to the acid and base chamber next to the BPM. Current utilization (ζ) is calculated from
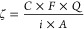
7where *F* is Faraday’s constant, *Q* is the volumetric flow rate, *i* is the current density, and *A* is the active area of the membrane. *C* is the molarity of H^+^ or OH^–^ produced in BPM at a certain current density calculated with the measured pH using the PHREEQC modeling package from the U.S. Geological Survey.^49^
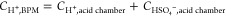
8
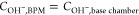
9

It is assumed in the calculation that the AEM membrane and Nafion membrane separating acid/base rinse chamber and acid/base chamber have perfect permselectivity—only SO_4_^2–^ is allowed to transport through AEM and only K^+^ is allowed to transport through Nafion. Therefore, *C*_K^+^_ in the acid chamber is kept constant at 0.8 M, and (*C*_SO_4_^2–^_ + *C*_HSO_4_^–^_) in the base chamber is kept constant at 0.4 M. The concentrations of H^+^, HSO_4_^–^, and SO_4_^2–^ in the acid chamber and the concentrations of OH^–^ and K^+^ in the base chamber are calculated with pH and charge balance. Higher than 88% current utilization was achieved in the base chamber and higher than 92% in the acid chamber for current densities up to 500 mA cm^–2^. This difference might be attributed to the proton exclusion of the AEM being less effective than hydroxide exclusion of Nafion, leading to the measured current utilization of acid being slightly higher than that of the base.

The electrochemical characterization of BPMs in equilibrium with 1 M H_2_SO_4_ next to the CEL and 1 M NaOH next to the AEL was carried out in the flow cell shown in Figure 2. Two reference electrodes were inserted into the cell, sitting at each side of the BPM during the four-electrode measurement. No Haber-Luggin capillary was used in this setup, so a 5.5 mm distance exists between the tip of the reference electrodes and the membrane surface. The resistances of the electrolytes across the 5.5 mm distance were measured by flowing the single kind of electrolyte through the cell without BPM in it and measuring the impedance between the two reference electrodes. A combined resistance of 5.45 ± 0.35 Ω cm^2^ was acquired for the electrolytes from 10 repeated measurements of impedance testing, which can cause a substantial potential drop and highlights the importance of iR correction used in this study.

**Figure 5:**
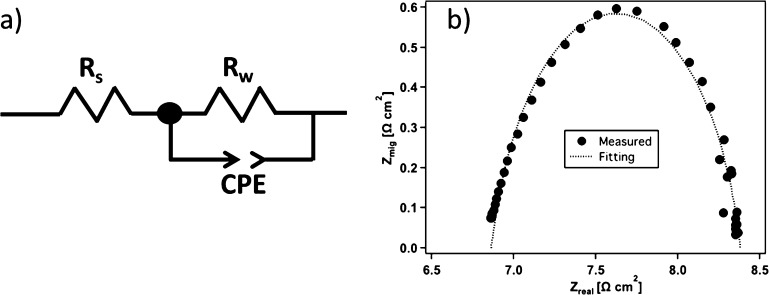
(a) Equivalent circuit used for fitting the EIS spectra in the ZView software; (b) measured EIS spectra and fitted data for an FBM at 20 mA cm^–2^.

The performance of water dissociation in the BPM junction was investigated via EIS analysis. While the voltage–current relationship provides limited information for water dissociation reaction in the BPM junction, EIS offers a more direct way to characterize this reaction. The first discussion of using AC impedance spectra to understand electric field enhanced water dissociation at the BPM junction was reported by Alcaraz et al. in 1996.^50−52^ Recently, this technique was used to differentiate electronic information in BPMs and to characterize the thickness of the space charge region.^53−55^Figure 5a shows the electrical equivalent circuit employed to analyze the EIS spectra in ZView software. The *R*_s_ value in the circuit is the sum of the resistance of the electrolytes and ion-exchange layers, and *R*_w_ is the resistance of water dissociation in the interfacial junction. The constant-phase element models the imperfect double layer and compensates for the nonhomogeneity in the BPM junction with a phase shift close to 1. When the frequency → ∞, *Z*_t_ → *R*_s_, and when the frequency → 0, *Z*_t_ → *R*_s_ + *R*_w_, with *Z*_t_ representing the total impedance associated with the overall circuit. An example of the Nyquist plot for a commercial FBM at 20 mA cm^–2^ is shown in Figure 5b. The equivalent circuit model shows good agreement with the measured results, with a goodness of fit of χ^2^ < 3 × 10^–4^.

**Figure 6:**
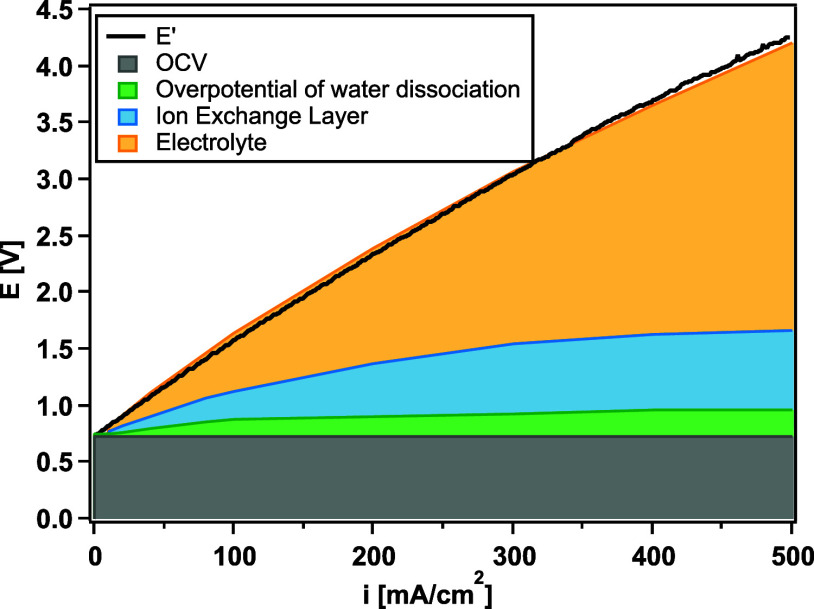
Voltage breakdown for FBM-calculated EIS data fitting. The solid line represents the measured voltage.

To understand the trend of the *V*–*I* curves, the voltage breakdown for the individual components and the entire electrochemical cell is presented in Figure 6. The black solid line is the measured voltage for the FBM with a current applied up to 500 mA cm^–2^. This line comprises the thermodynamic potential or open-circuit voltage, the overpotential for water dissociation in the BPM junction, and the ohmic drop through the electrolyte and the ion-exchange layers. It is often quoted that the standard thermodynamic potential of the BPM junction is calculated as^56,57^
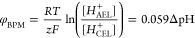
10where *R* is the universal gas constant 8.314 J mol^–1^ K, *T* is the temperature, and *F* is the Faraday constant 96,485 C mol^–1^. With 1 M H^+^ in the CEL and 1 M OH^–^ in the AEL to balance the fixed charges in the membrane, the thermodynamic potential to dissociate water is 0.83 V. However, phenomena such as the SWE and CRM help resupply ions to the interface, thereby weakening the inward diffusion forces and resulting in a weaker junction potential. The overpotential of water dissociation in the BPM junction and the voltage contributed from the ion exchange layers were calculated using the resistances from the fitted results of EIS. The voltage contributed from the electrolytes was measured in separate impedance tests in which a single kind of electrolyte (1 M H_2_SO_4_ or 1 M KOH) flew through the cell with no BPM. It is shown in Figure 6 that the calculated total voltage agrees well with the measured voltage. A considerable potential drop was caused by the electrolyte, accounting for more than 50% of the total voltage when the current density is higher than 300 mA cm^–2^, whereas the voltage attributed to water dissociation in the BPM junction is less than 30% of the total voltage. As a result, in the following sections, the reported voltages have been corrected to be electrolyte-free voltages unless otherwise specified in order to highlight the changes in water-dissociation rates that are not affected by the bulk electrolyte resistances.

### 3.2 GO_*x*_ Loading Effect in 2D BPM

**Figure 7:**
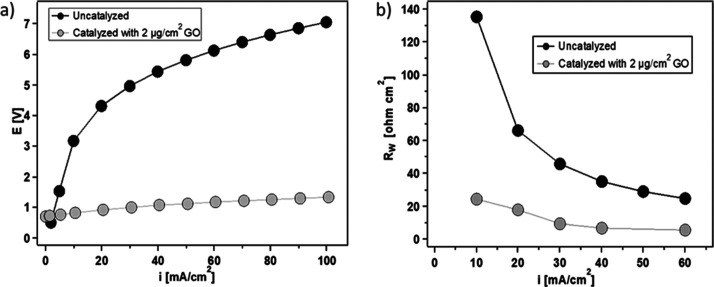
(a) *V*–*I* curves and (b) water-dissociation resistance of an uncatalyzed 2D BPM and a catalyzed 2D BPM with 2 μg cm^–2^ GO_*x*_.

Figure 7a shows the *V*–*I* characteristics of the uncatalyzed 2D BPM and 2D BPM catalyzed with 2 μg cm^–2^ GO_*x*_. The reduced voltage to achieve higher current densities for the GO_*x*_-catalyzed BPM indicates that GO_*x*_ in the BPM junction effectively promotes water dissociation. Compared with the control sample of an uncatalyzed 2D BPM, depositing only 2 μg cm^–2^ of GO_*x*_ in the BPM junction decreased the voltage by more than 75%, measuring 1.34 V at 100 mA cm^–2^. Correspondingly, the *R*_w_ at 60 mA cm^–2^ in the catalyzed 2D BPM decreased by more than 85%—from 40.4 to 5.0 Ω cm^2^—as illustrated in Figure 7b.

It was anticipated that with an increase of GO_*x*_ loading, additional active catalytic sites would be available for water dissociation, thereby decreasing the potential drop and *R*_w_. However, the catalytic effect of GO_*x*_ loading was found to plateau in the 2D BPM junction.

**Figure 8:**
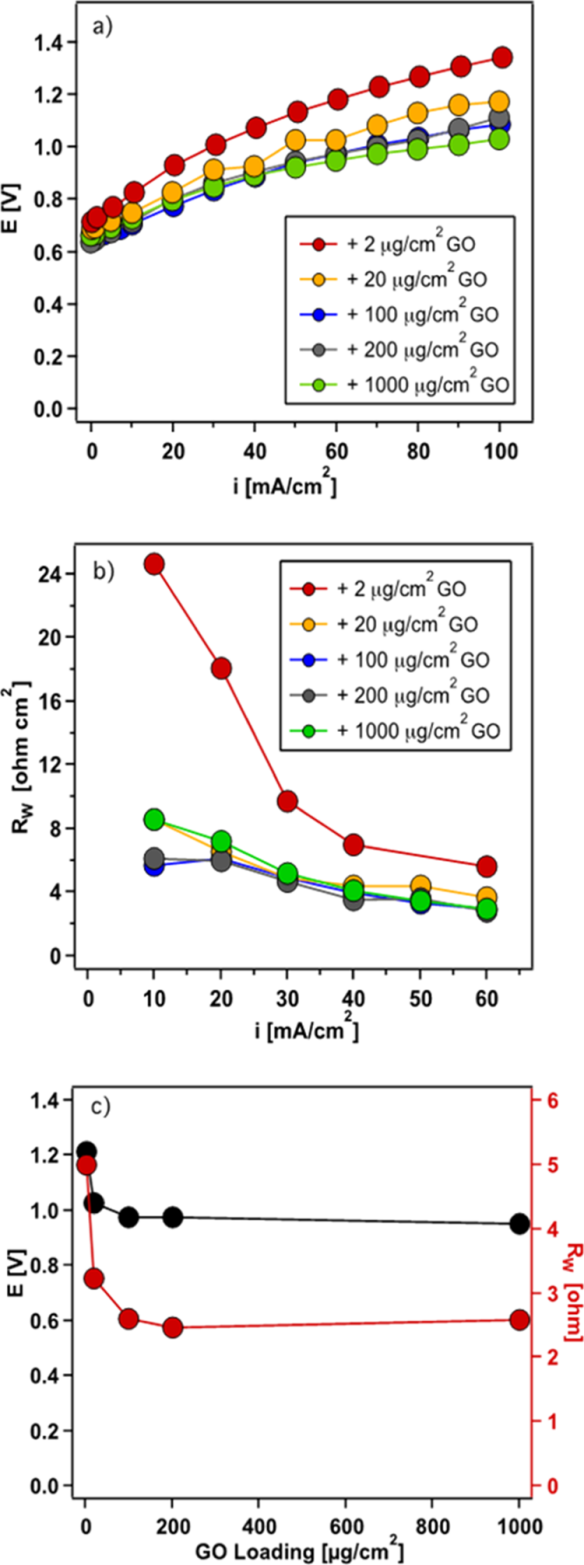
(a) *V*–*I* curves and (b) *R*_w_ of catalyzed 2D BPM. (c) Voltage (black circle) and *R*_w_ (red circle) at 60 mA cm^2^ as a function of the GO_*x*_ loading in the BPM junction.

To examine the effect of GO_*x*_ loading on the *R*_w_ in the BPMs, different GO_*x*_ loadings were deposited in the junction of 2D catalyzed BPMs using ultrasonic spray deposition, ranging from 2 μg cm^–2^ up to 1000 μg cm^–2^, with the corresponding *V*–*I* curves shown in Figure 8a. From 2 to 100 μg cm^–2^, the voltage decreased with increasing GO_*x*_ loading. However, at GO_*x*_ loadings above 100 μg cm^–2^ and up to 1000 μg cm^–2^, a similar performance was achieved. The *R*_w_ in 2D catalyzed BPMs with different GO_*x*_ loadings showed a similar trend, as shown in Figure 8a. *R*_w_ at 60 mA cm^–2^ decreased from 5 to 2.6 Ω cm^2^ when GO_*x*_ loading increased from 2 to 100 μg cm^–2^. An almost constant *R*_w_ was achieved for GO_*x*_ loadings of 100, 200, and 1000 μg cm^–2^. Figure 8c shows the comparison of the catalyzed 2D BPM with various GO_*x*_ loadings on their voltage drop and *R*_w_ at a current density of 60 mA cm^–2^. A sharp decrease in the voltage and *R*_w_ was achieved by increasing the GO_*x*_ loading to 100 μg cm^–2^, and the trend flattened out quickly. The optimal catalyst loading for 2D BPMs with full utilization of catalytic sites was obtained at 100 μg cm^–2^ GO_*x*_ loading.

**Figure 9:**
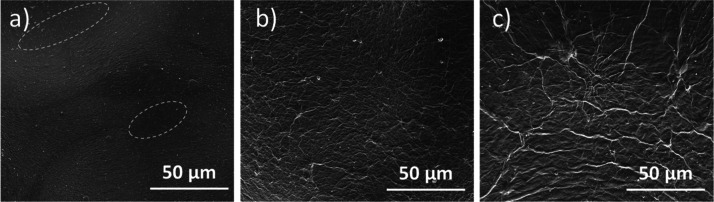
Top–down SEM images of 2D BPMs with GO_*x*_ loadings of (a) 2 μg cm^–2^, (b) 100 μg cm^–2^, and (c) 1000 μg cm^–2^.

To better understand the performance of the BPMs with different GO_*x*_ loadings, the morphology of the samples with different GO_*x*_ contents was compared by SEM images shown in Figure 9. It is evident from Figure 9a that the Nafion surface was not fully covered by GO_*x*_ at a relatively low loading (2 μg cm^–2^). With a GO_*x*_ loading of 100 and 1000 μg cm^–2^, the Nafion membranes were uniformly covered by filaments with similar elongated ridge structures. With an increase in the GO_*x*_ loading, the ridge structures showed lower density but larger size, and therefore, they had a rougher surface structure.

From comparing the morphology of the BPM surface as a function of loading in Figures 9a and 8b, it is possible that increasing the GO_*x*_ loading from 2 to 100 μg cm^–2^ leads to a variation in the fractional coverage of GO_*x*_ in the BPM junction. In this low-loading regime, additional GO_*x*_ leads to a higher density of more active sites for catalyzed water dissociation and a corresponding drop in voltage and *R*_w_ when GO_*x*_ loading is below 100 μg cm^–2^. After full surface coverage is achieved, further increasing the GO_*x*_ loading can then decrease the electric field in the junction because of an increased junction thickness and, therefore, lead to a lower water-dissociation rate. Although there appears to be marginal performance improvement when GO_*x*_ loading exceeds 100 μg cm^–2^ (Figure 8a), the BPMs tend to delaminate much easier when GO_*x*_ loading is greater than 100 μg cm^–2^ because of the lack of firm bonding between the two layers. Thus, an optimal GO_*x*_ loading of 100 μg cm^–2^ was chosen for fabricating 2D BPMs.

### 3.3 Comparison of Novel GO_*x*_-Catalyzed 2D and 3D BPMs with Commercial BPMs

**Figure 10:**
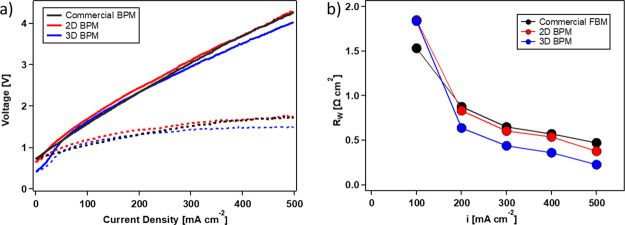
(a) *V*–*I* curves and (b) *R*_w_ of fabricated 2D BPM with 100 μg cm^–2^ GO_*x*_, 3D BPM with 100 μg cm^–2^ GO_*x*_, and commercial BPM FBM. Solid lines represent measured voltage and dashed lines represent electrolyte-free voltage.

To compare the electrochemical performance of the above fabricated BPMs with a commercial BPM, galvanodynamic scans were performed up to 500 mA cm^–2^ at 1 mA cm^–2^ s^–1^ for the 2D BPM, 3D BPM, and FBMs. The 2D BPM and 3D BPM were both catalyzed with 100 μg cm^–2^ of GO_*x*_. *V*–*I* curves in Figure 10a show that no limiting current density caused by water depletion in the BPM junction was encountered up to 500 mA cm^–2^, indicating sufficient water transport through the ion-exchange layers.

Compared to a commercial BPM (FBM), the 2D BPM shows slightly higher voltages (maximum 0.13 V higher) at current densities below 400 mA cm^–2^. When the current density exceeds 400 mA cm^–2^, the voltage of the 2D BPM almost overlaps with the FBM. Figure 10b shows similar *R*_w_ for the 2D BPM and FBM; however, at 500 mA cm^–2^, the 2D BPM shows 0.1 Ω cm^2^ lower resistance than the FBM. This decrease could indicate a higher catalytic activity and water-dissociation efficiency in the 2D BPM than in the FBM. However, because the exact composition and structure of FBM is unknown, it is difficult to conclude whether the difference is directly or dominantly because of the catalyst loading or active area.

The 3D BPM made by electrospun fibers and catalyzed with 100 μg cm^–2^ GO_*x*_ showed both the lowest voltage and lowest *R*_w_ for current densities higher than 200 mA cm^–2^. At 500 mA cm^–2^, the electrolyte-free voltage for the 3D BPM was 0.25 V lower compared to the 2D BPM and FBM. Although the same catalyst loading was deposited in the 2D BPM and 3D BPM, the better performance of the 3D BPM is likely due to the combined effect of the GO_*x*_ catalyst along with the larger interfacial contact area of the interface, which leads to lower local water dissociation current densities. For the 3D BPM with a 5 μm thickness of the 3D co-electrospun junction, the effective contact area is about 80 times greater than the calculated geometric area based on an average fiber diameter of 120 nm. At the same time, the intertwined Nafion/PFAEM fibers provide unobstructed pathways for the water-dissociation products (H^+^ and OH^–^) to migrate away from the junction quickly with less chance of recombination, which therefore further promotes water-dissociation efficiency.

### 3.4 Stability Testing

So far, only a few previous studies^28^ have addressed BPM performance and stability at current densities higher than 150 mA cm^–2^. However, with the increasing number of studies using BPMs in MEAs where high current densities are expected, it is critical to understand the membrane stability at high current densities.

**Figure 11:**
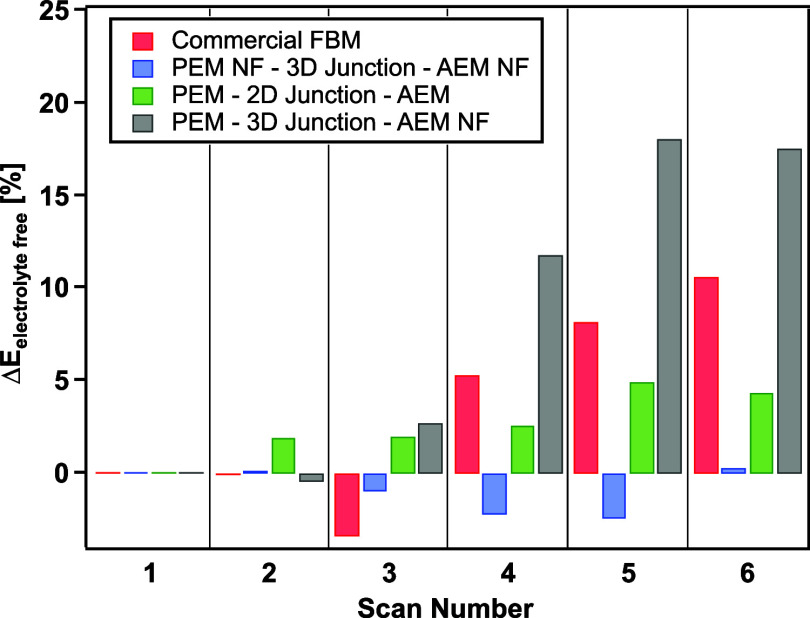
Voltage change (electrolyte-free) at 500 mA cm^–2^ during repeated galvanodynamic scans from 0 to 500 mA cm^–2^.

**Figure 12:**
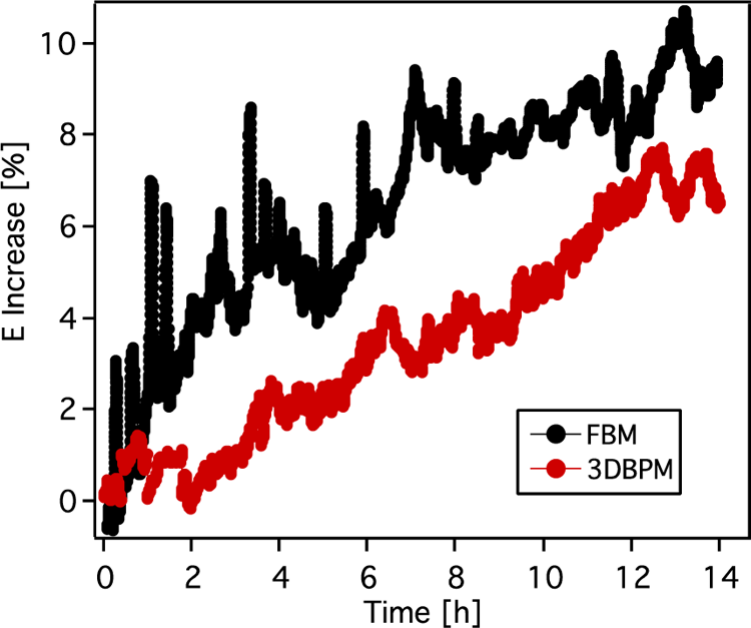
Voltage increase during galvanostatic testing at 500 mA cm^–2^ for 14 h for FBM and electrospun 3D BPM.

The electrochemical stability of fabricated BPMs and a commercial FBM was evaluated with repeated galvanodynamic scans from 0 to 500 mA cm^–2^. The voltage changes (electrolyte-free) at 500 mA cm^–2^ with the scan number are reported in Figure 11. After six scans, the electrolyte-free voltage of FBM increased 11%, indicating degradation of the membrane. The 2D BPM showed only 5% voltage increase at the end of repeated scans, suggesting significantly better stability than the FBM. Making a 2D BPM by hot-pressing preformed membranes together has always been a challenge^27^ because of easy delamination and blistering. However, in this study, the 2D BPMs made with ionomers of similar perfluorinated structure showed better durability than the commercial FBM, which is mechanically reinforced with woven PEEK. For the 3D BPM, a very small variation of the voltage change was observed during the repeated scans, and only <1% voltage increase was reached after six scans. It is shown in Figure 12 that when holding at 500 mA cm^–2^ for 14 h, the voltage of 3D BPM increased by 7.5%, while the voltage of FBM increased more than 10%. These tests highlight that the 3D BPM has improved stability at current densities up to 500 mA cm^–2^, which could be explained by the interlocking of Nafion and PFAEM fibers at the 3D co-electrospun junction, preventing membrane delamination and degradation.

In addition to the previously mentioned BPMs, we examined a hybrid BPM made by electrospinning the catalyzed 3D junction and PFAEM fibers on top of a Nafion membrane (PEM–3D junction–AEM NF). A similar BPM was studied by Hohenadel and co-workers^29^ where the BPM was composed of an electrospun 3D junction catalyzed with Al(OH)_3_ between two casted ion exchange layers. Their BPM showed excellent performance; however, the stability was not investigated. Interestingly, this hybrid BPM was found to be the least stable under repeated scans, showing an 18% voltage increase at 500 mA cm^–2^ at the end of the sixth scan. Similar to the 3D BPM, this hybrid BPM also has a 3D co-electrospun junction. The only difference between the hybrid BPM and the 3D BPM was the CEL structure: the hybrid BPM used a preformed Nafion membrane and the 3D BPM used electrospun Nafion fibers. It is possible that the severe degradation in the hybrid BPM comes from the delamination between the Nafion membrane and the electrospun layer. Even though both the 3D BPM and hybrid BPM experienced the same vapor exposure and hot-pressing procedure, the bonding from the intertwined and interlocked structure between the layers in the 3D BPM is fundamentally higher than the bonding between the electrospun fibers and a preformed membrane in the hybrid BPM. This result demonstrates that the superior stability in the 3D BPM comes not only from the 3D co-electrospun junction but also from the strong bonding between each layer throughout the whole membrane structure.

## 4 Conclusions

In summary, GO_*x*_-catalyzed 2D BPMs and 3D BPMs were fabricated and their electrochemical performance under reverse bias was evaluated. The effect of GO_*x*_ loading on reducing *R*_w_ in the 2D BPM saturated around 100 μg cm^–2^, at which GO_*x*_ fully covered the junction area. The catalyzed 2D BPM showed comparable performance with a commercial FBM up to 500 mA cm^–2^ without reaching a limiting current density of water depletion in the junction. A novel electrospun 3D BPM showed even better performance than the FBM and the 2D BPM, achieving *E*_iR-free_ of 1.5 V at 500 mA cm^–2^, which can be attributed to the significantly increased area of the catalytic sites from the dual-fiber co-electrospun 3D junction. The entangled fibers of the opposite fixed charge facilitate the migration of water-dissociation products away from the junction, further reducing *R*_w_ by decreasing the possibility of water recombination. The intertwined fibers throughout the membrane, especially in the 3D junction, prevent the formation of blistering or membrane delamination when operating at high current densities, suggesting that they may be suitable in an MEA configuration for applications in CO_2_R, water electrolysis, fuel cells, and other electrochemical applications.
